# The different impact of a high fat diet on dystrophic mdx and control C57Bl/10 mice.

**DOI:** 10.1371/currents.RRN1276

**Published:** 2011-11-15

**Authors:** Hannah G Radley-Crabb, Marta L Fiorotto, Miranda D Grounds

**Affiliations:** ^*^School of Anatomy and Human Biology, the University of Western Australia, Perth, Australia and ^†^USDA/ARS Children’s Nutrition Research Center, Department of Pediatrics, Baylor College of Medicine, Houston, Texas, USA.

## Abstract

The absence of functional dystrophin protein in patients with Duchenne muscular dystrophy (DMD) and dystrophic mdx mice leads to fragile myofibre membranes and cycles of myofibre necrosis and regeneration. It is proposed that both DMD patients and mdx mice have an altered metabolism and impaired energy status and that nutritional supplementation may reduce the severity of dystropathology. This research compares the in vivo responses of dystrophic mdx and normal control C57Bl/10 mice to a high protein (50%) or a high fat (16%) diet. Consumption of a high protein diet had minimal effects on the body composition or muscle morphology in both strains of mice. In contrast, differences between the strains were seen in response to the high fat diet; this response also varied between mdx mice aged <24 weeks, and mdx mice aged 24 - 40 weeks. C57Bl/10 mice demonstrated many negative side effects after consuming the high fat diet, including weight gain, increased body fat, and elevated inflammatory cytokines. In contrast, after consuming the high fat diet for 16 weeks the mdx mice (< 24 weeks) remained lean with minimal fat deposition and were resistant to changes in body composition. These results support the proposal that energy metabolism in dystrophic mdx mice is altered compared to normal C57Bl/10 mice and this enables the mdx mice to better metabolise the high fat diet and avoid fat deposition. However, older mdx mice (24 – 40-week-old), with increased energy intake, exhibited some mild adverse effects of a high fat diet but to a far lesser extent than age-matched C57Bl/10 mice. Benefits of the high fat diet on dystrophic muscles of young mice were demonstrated by the significantly increased running ability (km) of voluntarily exercised mdx mice and significantly reduced myofibre necrosis in 24-week-old sedentary mdx mice. These novel data clearly identify an ‘altered’ response to a high fat diet in dystrophic mdx compared to normal C57Bl/10 mice. Our data indicate that the high fat diet may better meet the energy needs of mdx mice to reduce muscle damage and improve muscle function.

## Introduction

Duchene muscular dystrophy (DMD) is characterised by progressive muscle weakness and wasting resulting from a lack of functional dystrophin that promotes increased myofibre membrane fragility, repeated cycles of myofibre necrosis and regeneration, and the eventual replacement of skeletal muscle by fatty and fibrous connective tissue.  The specific mechanism(s) responsible for myofibre necrosis are still unclear, although there are strong associations with excessive inflammation, increased intracellular calcium levels, elevated oxidative stress, and metabolic abnormalities [1] [2] [3] [4] [5] [6] [7] [8]. Corticosteroids remain the standard pharmacological treatment [9] [10] [11] [12] although promising approaches to replace the defective dystrophin gene have been extensively investigated over the last 10 years [13] [14] [15] [16] [17]. Clearly, studies to identify strategies that will mitigate the muscle damage and sustain efficient repair are warranted.

The mdx mouse is a widely used animal model for pre-clinical DMD research despite there being fundamental differences in growth parameters, body size and muscle loading, that lead to significant differences in disease severity between dystrophic mdx mice (C57Bl/10ScSn^mdx/mdx^) and DMD patients [Reviewed in [18] [19] [20] [21]]. Studies in both DMD patients and mdx mice indicate that dystrophin defects may also lead to altered skeletal muscle metabolism and an impaired energy status. Repeated cycles of myofibre necrosis and regeneration, increased demand on the sarcoplasmic reticulum to regulate intracellular calcium, defective mitochondrial function, increases in both protein synthesis and protein degradation rates, altered oxidative stress and disruption in nNOS signalling may all contribute to an altered metabolic state[4] [22] [23] [24] [25] [26] [27] [28] [29] [30]. In support of an altered metabolism in dystrophic muscle, 48 hours of fasting significantly increased myofibre necrosis in muscles from the hind limb and lumbar region of 6-month-old mdx mice (yet no change in muscle morphology of control C57BL/10 mice), suggesting a strong dependence on an adequate energy intake to maintain dystrophic muscle structure [31]. 

Dietary interventions in the form of various amino acids (with different biochemical effects) or other nutritional supplements have shown variable beneficial effects in both mdx mice and DMD patients [Reviewed in [7] [8] [32] [33] [34]. Recently completed clinical trials have examined the safety and role of specific amino acid supplementation (creatine and glutamine) in DMD patients and demonstrated some beneficial effects [35] [36]. In mdx mice, a creatine enriched diet (10% w/w in chow) fed to new-borns (via lactating mothers) strongly reduced the onset of muscle necrosis in the fast-twitch EDL muscle and also improved mitochondrial respiration capacity [25]. In addition, creatine, taurine and glutamine treatments have shown various benefits in treadmill-exercised adult mdx mice [37] [38]. A combined nutritional therapy (creatine monohydrate, conjugated linoleic acid, α-lipoic acid, and β-hydroxy-β-methylbutyrate) administered, in addition to prednisolone, for 8 weeks increased muscle strength and reduced the extent of dystropathology in 12-week-old treadmill-exercised mdx mice [39].  A combination of taurine (1g/(kg bw.day) - orally) and prednisolone (1mg/(kg bw.day) - i.p. injection) treatment of treadmill-exercised mdx mice (4-8 weeks of age) also markedly improved forelimb grip strength, compared to either taurine or prednisolone treatment alone [40]. In dystrophic laminin deficient (129ReJ dy/dy) mice, a high protein diet (50%) improved muscle morphology and caused a shift to a more ‘normal’ protein metabolism [41].  In many cases however, the molecular/metabolic basis for the variable benefits reported for the different interventions in dystrophic skeletal and heart muscle remains to be determined.  Additional, more mechanistically based research will be required to establish optimal dietary interventions to reduce the severity of dystropatholgy and maintain muscle function for potential application to DMD. 

In a preliminary study that tested the effects of a high fat diet on the dystrophic mdx heart, Hoey et al (2005, data unpublished) reported a striking difference in the bodyweights of dystrophic mdx and control male mice fed a high fat diet (~15% w/w) for 9 weeks (from 6–15 weeks of age). The body weight of mdx mice was unaffected by the change in diet, whereas the control C57Bl/10 mice showed significantly greater body weight and % body fat, as expected. The dystropathology of skeletal muscles was not examined in this study, but these results led us to propose that due to their altered energy flux, a high fat diet may reduce the extent of dystropathology and may be metabolically beneficial to mdx mice. 

The aims of the present study were to compare in sedentary adult and voluntarily exercised C57Bl/10 and mdx mice the effects of a high protein and high fat diet on the body composition, muscle morphology and dystropathology (for mdx mice only), and expression levels of genes that play pivotal roles in the metabolism, inflammation [1] [6] [42] [43], adiposity [Reviewed in [44] [45] [46]] and remodelling of the skeletal myofibre [Reviewed in [46] [47] [48]]. 

## Methods

**Animals.** Experiments were conducted on male non-dystrophic (control) C57Bl/10ScSn and dystrophic mdx (C57Bl/10ScSn^mdx/mdx^) mice (hereafter referred to as C57 and mdx); all mice were obtained from the Animal Resource Centre, Murdoch, Western Australia. They were maintained at the University of Western Australia on a 12-h light/dark cycle, under standard conditions, with free access to food and drinking water. All animal experiments were conducted in strict accordance with the guidelines of the National Health and Medical Research Council Code of practice for the care and use of animals for scientific purposes (2004), and the Animal Welfare act of Western Australia (2002), and were approved by the Animal Ethics committee at the University of Western Australia.

**Experimental groups and custom diets. **This study consisted of the following 3 groups of mice, n=8 mice for all groups. **Group 1)** sedentary C57 and mdx mice fed a custom diet from 8-24 weeks of age, **Group 2) **sedentary C57 and mdx mice fed a custom diet from 24-40 weeks of age and **Group 3)** voluntarily exercised mdx mice fed a custom diet from 8-12 weeks of age. All mice were fed a standard (cereal-based) mouse chow (meat free, 5% fat, 19% protein) prior to the study. The three customised semi-purified diets; Control (7% fat, 19% protein - AIN93G), High Fat (16% fat, 19% protein -  SF 06-040) and High Protein (7% fat, 50% protein – SF 00-252) were manufactured (all in pellet) form by Specialty Feeds Glen Forest Western Australia www.specialtyfeeds.com.au (Table 1). Pilot trials were conducted prior to commencement of the study to ensure that all diets were palatable. Throughout the study mice were caged in groups of 4-8 and group body weights were measured weekly, mice were not tracked individually for the duration of the study. Group food intake was monitored twice weekly by subtracting from a pre-weighed amount, the food that remained in the food compartment on the cage lid, and food inside the cage that could be separated from the cage bedding. Energy intake was calculated by adjusting food consumption (g) to metabolisable energy content of each diet (Table 1): metabolisable energy content was calculated according to guidelines of Food and Agriculture Association of the United Nations, http://www.researchgate.net/journal/0254-4725_FAO_food_and_nutrition_paper.  

**Figure:**
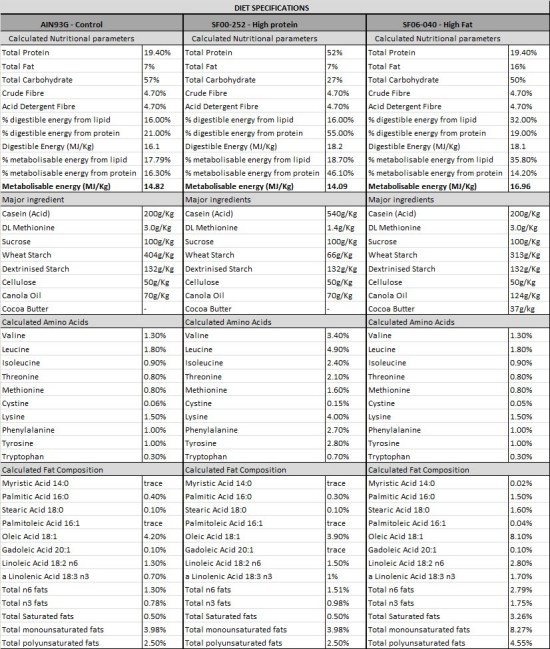
**Table 1. Diet specifications for the 3 semi-pure diets used in this study: control (AIN93g), high protein (SF00-252) and high fat (SF06-40).** Energy intake was calculated based on the metabolisable energy content of each diet. All diets were manufactured by Specialty Feeds, Glen Forrest, WA.  

**Voluntary exercise (mdx mice only).** The low level of muscle damage in dystrophic adult mdx mice can be exacerbated by voluntary exercise [18] . Mdx mice were caged individually with free access to a voluntary running wheel for 4 weeks (from 8 to 12 weeks of age). Exercise data were collected via a small magnet attached to the mouse wheel, and a sensor from a bicycle pedometer attached to the back of the cage. The pedometer records single wheel revolutions, allowing total distance (km) run by an individual mouse to be determined, as per  [1] [49] [50]. The mice run the most during the night because they are normally nocturnal [50] [51]. Throughout the experiment the mice were monitored daily for food consumption and distance run. 

**Tissue collection. **Mice were killed at either 12, 24 or 40 weeks of age by cervical dislocation, while under terminal isoflurane (Bomac Australia) anaesthesia (2%v/v). Total body weight, epididymal fat pad weight, and gastocnemius muscle weight were recorded immediately. Blood serum was collected via cardiac puncture, the gastrocnemius muscle was snap frozen in liquid nitrogen for gene expression analysis and the quadriceps and tibialis anterior (TA) muscles were collected for histology. 

**Skeletal muscle histology.** The quadriceps muscles were dissected and immediately fixed in 4% paraformaldehyde (Sigma P6148) for 48 hours. Muscles were then placed into 70% ethanol, processed in a Shandon automatic tissue processor overnight, and finally paraffin embedded for sectioning. Transverse sections (5 μm) were cut through the mid-region of each muscle on a microtome. Slides were stained with Haematoxylin and Eosin (H&E) for morphological analysis of dystropathology (e.g. myofibre necrosis and adipocyte content) as per the TREAT-NMD recommended standard protocol “Histological measurements of dystrophic muscle - M.1.2_007” http://www.treat-nmd.eu/research/preclinical/SOPs/.  The TA muscles were collected, and embedded in tragacantch gum (Sigma Aldrich G1128) mounted on a small cork block, and quenched in isopentane cooled in liquid nitrogen. Muscles were stored at -80ºC until cut at 5μm on a cryostat. TA sections were stained with Sirius red to distinguish myofibres (yellow) from collagenous myofibre membranes (red), and which allows for easy and accurate quantification of myofibre cross-sectional area (CSA).  Distinct areas of active myofibre necrosis and very early muscle regeneration (small myotubes) were not included in myofibre cross sectional area analysis.  

**Image capture and Histological image analysis.** Non-overlapping tiled images of transverse muscle sections provided a picture of the entire muscle cross section. Images were acquired using a Leica DM RBE microscope equipped with a Hitachi HVC2OM digital camera, Image Pro Plus 4.5.1 software, and Vexta stage movement software. Tiled images were taken at 10x magnification. Histological analysis was carried out on whole cross sections of the quadriceps muscle (dystropathology and adipocyte content) and the frozen TA muscle (myofibre CSA). Muscle morphology was selectively drawn by the researcher using Image Pro Plus 4.5.1 software.  

**Serum Creatine Kinase Assay.** While under terminal anaesthesia blood from the mdx mice was collected via cardiac puncture. Blood was refrigerated overnight, centrifuged for 3 min (1200 rpm) and serum removed. Blood serum creatine kinase (CK) analysis was completed at the Murdoch Veterinary Hospital, Murdoch, WA. 

**Quantification of Gene expression (relative to L-19).** Consumption of the high fat diet induced many changes in the body composition of both C57 and mdx mice, whereas the high protein diet did not. Therefore gene expression was quantified only in mice which consumed the control or high fat diet.  RNA was extracted from snap frozen gastrocnemius muscles, DNAse treated, reverse transcribed into cDNA and purified. RT-PCRs were run on a Corbett 3000 (Corbett Research) using QIAGEN quantifast SYBR green PCR mix and QIAGEN Quantitect Primer Assays for TNF (QT00104006), IL-1β (QT01048355), IL-6 (QT00098875), PPAR alpha (QT00137984), PPAR delta/beta (QT00166292), PPAR gamma (QT00100296), PGC-1α (QT00095578)  and standardised to an appropriate house-keeping gene; ribosomal protein L-19 (QT01779218) as per [52]. mRNA expression was calculated and standardised using Roto-gene 6.1 and Microsoft Excel software.  

**Statistical analysis**. Analysis was completed using Microsoft Excel and SPSS 16.0. All variables were analysed by ANOVAs (to account for diet, strain, age, and exercise) and Least Significant Difference (LSD) post-hoc tests. All data are expressed as mean + SEM.  

## Results

### Food consumption 

There were no significant differences in average food intake (g/day) in either strain of sedentary mice (8-24 or 24-40-week-old) across the 3 custom diets. Additionally, there were no significant differences in average daily food consumption adjusted for differences in body weight [g/ (g bw. day)] across the 3 custom diets for both strains of 8 - 24-week-old mice (Figure 1A). However, there was a significant decrease in food consumption [g/ (g bw. day)] with age in both strains of mice and this decrease was significantly greater for 24-40-wk-old C57 mice on the high fat diet compared to both the control and high protein diets (Figure 1A).  Daily energy intakes (kJ/day) averaged over the entire 16 week period  were decreased significantly in both strains of 8 - 24-week-old mice on the high protein diet (Figure 1B). The trend for increased energy intakes (kJ/day) in both strains of mice on the high fat diet at 8–24 weeks of age compared to mice on the control diet did not attain statistical significance.  In the older mice (24-40 weeks) on the high fat diet there was a significant increase in average daily energy intake (kJ/day) in both strains compared to mice on either the control or high protein diet (Figure 1B).  When adjusted for body weight, there were no differences in energy intake [kJ/ (g bw .day)] between 8 – 24-week-old C57 or mdx mice on the control diet; however, both C57 and mdx mice on the high protein diet consumed significantly less compared to mice on either the control or high fat diet (Figure 1C). Adjusted energy intakes in both strains of mice decreased significantly with age, and 24 - 40-week-old mdx mice on the high fat diet consumed significantly (P<0.02) more energy [kJ / (g bw .day)] than all other groups of mice (Figure 1C).  

**Figure:**
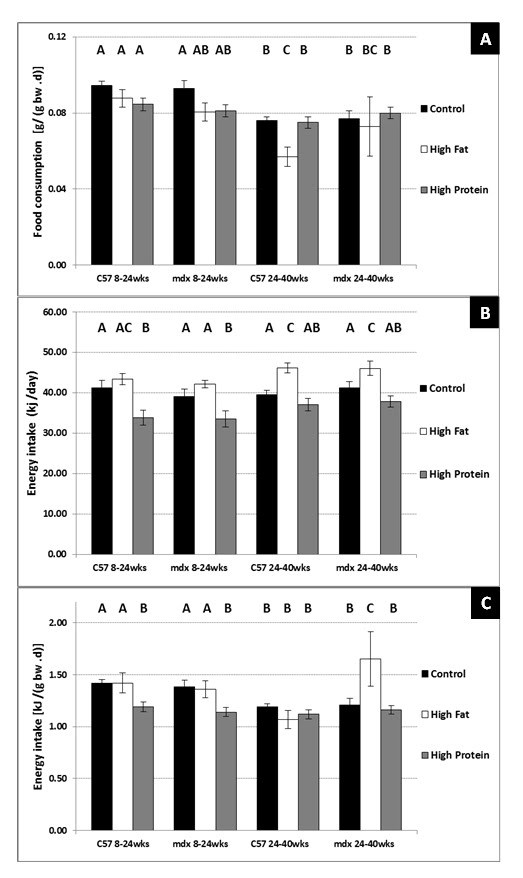
**Figure 1. Average daily food consumption [g/ (g bw .d)] (A), absolute energy intake (kJ/ day) (B) and standardised energy intake [kJ/ (g bw .d)] (C), for C57 and mdx mice; **a comparison of sedentary mice on a control diet, high fat diet or high protein diet between 8-24 weeks of age and 24-40 weeks of age. Bars represent standard error. N= 8 for all groups. A,B,C denotes significant differences, groups with different letters are significantly different from each other (P<0.05).

### Body Composition 

**Group 1) 8 – 24-week-old sedentary mice. **The average body weight of the mdx mice at the beginning of the study (8-week-old) that previously had been fed a standard (cereal-based) mouse chow was significantly heavier (P=0.02) than that of age-matched C57 mice (C57 21.6 ^+^/_- _0.23 g vs. mdx 23.4 ^+^/_- _0.72 g). This difference in body weight was maintained throughout the study until 24 weeks of age for mice on the control and high protein diet (Figure 2A).  After consuming the high fat (HF) diet for 16 weeks, the final bodyweight of 24-week-old C57 mice was significantly greater (P=0.04) than for C57 mice on the control (C) diet. In contrast, there was no difference in the bodyweight of 24-week-old mdx mice fed the high fat or control diet (Figure 2A). The largest gain in body weight (group average) between 8 and 24 weeks of age was seen in the C57 mice fed the high fat diet. The average changes in body weight for C57 mice (% change of sample group) were 130% after control diet, 140% after high protein diet and 159% after high fat diet. The average changes in body weight for mdx mice (% change of sample group) were 130% after control diet, 132% after high protein diet and 137% after high fat diet.

**Figure:**
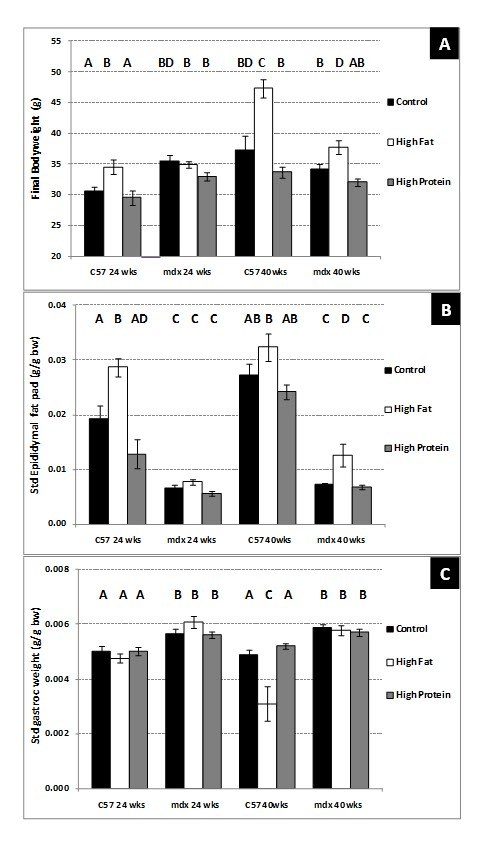
**Figure 2. Composition of sedentary 24 and 40-week-old C57 and mdx mice; **a comparison of sedentary mice on a control diet, high fat diet or high protein diet. (A) Total body weight. (B) Standardised epidiymal fat pad weight (g fat/g bw). (C) Standardised gastrocnemius muscle weight (g muscle /g bw). Bars represent standard error. N= 8 for all groups. A,B,C,D denotes significant differences, groups with different letters are significantly different from each other (P<0.05).

Epididymal fat pad weight increases in male C57Bl/6J mice after consumption of a high fat diet [53] and was measured in this study as an indicator of obesity. After consuming the control diet, the epididymal fat pad weight adjusted for body weight (g fat /g bw) was significantly (P<0.001) heavier in C57 compared to mdx mice at 24 weeks of age (Figure 2B). The high protein diet had no significant impact on epididymal fat pat weights in either strain of mice; fat pads remained heavier in C57 mice compared to mdx mice.   After consuming the high fat diet, the epididymal fat pad weight adjusted for body weight was significantly (P=0.015) higher in C57 mice compared to C57 mice fed the control diet (Figure 2B) and, at sacrifice, large amounts of adipose tissue were conspicuous around the sternum, kidneys and hip joints. In contrast, 24-week-old mdx mice fed a high fat diet were very lean and had small epididymal fat pads, similar in weight to mdx mice fed the control or high protein diet (Figure 2B). In the abdominal cavity, the epididymal fat pads often of mdx mice could not be seen until the testes were pulled up into the abdominal cavity. 

Gastrocnemius muscle weight (g muscle /g bw) was significantly (P=0.04) heavier in mdx mice fed a control diet compared to C57 mice at 24 weeks of age (Figure 2C). Absolute gastrocnemius muscle weight was also significantly (P<0.05) heavier in mdx mice at this age (C57 0.152g ^+^/_-_ 0.002 vs. mdx 0.190g ^+^/_-_ 0.004). Neither the high protein nor high fat diet resulted in any differences in standardised or absolute gastrocnemius muscle weight in either strain of mice.  

**Group 2) 24 – 40-week-old sedentary mice. **There was no significant difference in the body weights of 40-week-old C57 mice and mdx mice on the control diet (Figure 2A); nor did the high protein diet have any significant effect of the body weight of either strain of mice (Figure 2A). However, after consuming the high fat diet for 16 weeks, the average body weight of 40-week-old C57 mice was significantly (P<0.01) greater (by approximately 10g) compared to that of C57 mice on the control diet. Consumption of the high fat diet also significantly (P=0.04) increased the body weight of the older mdx mice (Figure 2A). The largest increase in body weight (group average) was seen in C57 mice on the high fat diet.  The average changes in body weight for C57 mice (% change of sample group) were 116% after control diet, 102% after high protein diet and 141% after high fat diet. The average changes in body weight for mdx mice (% change of sample group) were 103% after control diet, 94% after high protein diet and 110% after high fat diet.       

On the control diet the average epididymal fat pad weight adjusted for body weight (g/g bw) was significantly (P<0.01) greater in 40-week-old C57 mice compared to mdx mice (Figure 2B). The high protein diet had no impact on the epididymal fat pad weight in either strain of the older mice. On the high fat diet, there was a slight increase (but not significant P=0.38) in body weight-adjusted epididymal fat pad weight of C57 mice (Figure 2B). The absolute epididymal fat pad weight was significantly (P=0.03) increased (HF 1.42g ^+^/_- _0.06 vs. C 1.16g ^+^/_- _0.2) and observations made during tissue collection showed very large fat pads and pronounced adipose tissue around the sternum, kidneys, intestines and hip joints. The 40-week-old mdx mice were still very lean with no change in the body weight adjusted epididymal fat pad weight between 24 and 40 weeks of age for mdx mice fed a control diet (Figure 2B). In contrast to the younger 8 – 24-week-old mdx mice, the high fat diet significantly (P=0.035) increased epididymal fat pad weight in 40-week-old mdx mice (Figure 2B), although to a much lesser extent than C57 mice. 

Gastrocnemius muscle weight adjusted for bodyweight was significantly (P=0.045) heavier in mdx mice fed a control diet compared to C57 at 40 weeks of age (Figure 2C). Absolute gastrocnemius muscle weight was also significantly (P<0.05) heavier in mdx mice at this age (C57, 0.178 ^+^/_- _0.003 g vs. mdx, 0.198 ^+^/_- _0.005 g). There was no change in gastrocnemius muscle weight adjusted to bodyweight in mdx mice between 24 and 40 weeks of age. However there was a significant (P<0.05) increase in absolute gastrocnemius muscle weight with age in C57 mice (24 wk, 0.152^+^/_- _0.002 g vs. 40wk 0.178 ^+^/_- _0.003 g). The high protein diet had no effect on the muscle weights of either strain.The high fat diet caused no change in absolute gastrocnemius muscle weight in either strain of mice (data not shown), but due to the large increase in body weight in C57 mice after consuming a high fat diet standardised gastrocnemius muscle weight in C57 mice was reduced significantly (P=0.01) (Figure 2C). There was no effect of the high fat diet on the standardised gastrocnemius muscle weight in mdx mice. 

### Myofibre Size (control diet only) 

There was no difference in the average myofibre CSA in the tibialis anterior (TA) muscle from 12-week-old C57 and mdx mice on the control diet (Figure 3). Between 12 and 24 weeks of age, myofibres in the mdx TA continued to grow (hypertrophy) and were significantly larger on average than C57 myofibres at 24 weeks of age (P=0.02) (Figure 3).  Dystrophic myofibres do not continue to hypertrophy and average myofibre CSA in 40-week-old mdx mice was significantly reduced (P=0.025) compared to 24-week-old mice and not different from the average mdx myofibre CSA at 12 weeks of age. There was no change in myofibre CSA between 12, 24, and 40-week-old C57 mice.

**Figure:**
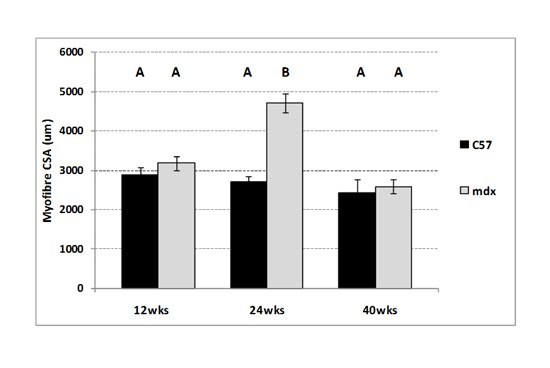
**Figure 3. Myofibre cross-sectional area in 12, 24 and 40-week-old sedentary C57 and mdx mice. **Bars represent standard error. N= 6 mice for all groups (at least 500 myofibres measured per mouse). A,B denotes significant differences, groups with different letters are significantly different from each other (P<0.05).

### Muscle morphology and dystropathology

**Group 1) 8 – 24-week-old sedentary mice.**  Myofibre necrosis (% CSA) was significantly (P=0.04) decreased in the quadriceps muscle of 24-week-old mdx mice fed the high fat diet and was approximately half the amount present in mice fed, the control diet (Figure 4A). The high protein diet had no effect on myofibre necrosis (compared to control diet). Myofibre necrosis is not a feature of control C57 mice and therefore it was not measured. There were no significant differences in adipocyte content of the quadriceps muscle in 24 week old C57 and mdx mice after consuming any of the 3 diets.  

**Figure:**
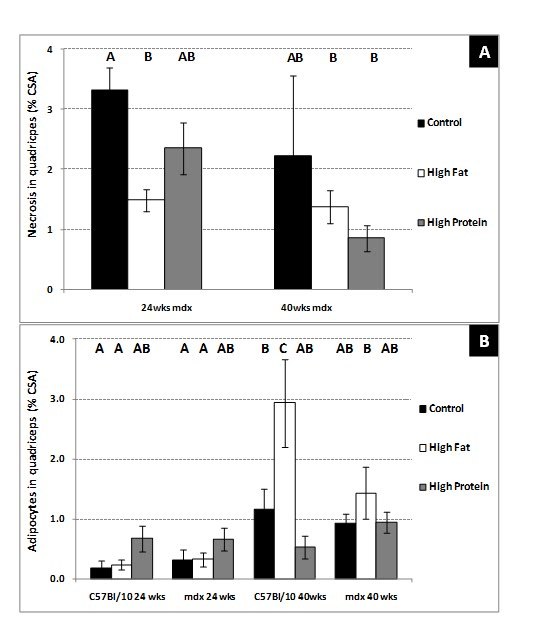
**Figure 4. Myofibre necrosis in sedentary mdx mice and adipocyte content of both C57 and mdx mice aged 24 and 40 weeks; **a comparison of sedentary mice on a control diet with mice on either a high fat or high protein diet. (A) Myofibre necrosis in the quadriceps muscle (mdx only). (B) Adipocyte content in the quadriceps muscle of both strains. Bars represent standard error. N= 8 for all groups. A,B,C denotes significant differences, groups with different letters are significantly different from each other (P<0.05).

**Group 2) 24 – 40-week-old sedentary mice.** There was no change in the level of myofibre necrosis in the quadriceps muscle between 24 and 40 week old mdx mice (Figure 4A) and no effect of either the high protein or high fat diet on myofibre necrosis in 40-week-old mdx mice (Figure 4A). The high protein diet had no effect on the adipocyte content of the quadriceps muscle in either strain of 40-week-old mice. Adipocyte content of the quadriceps increased significantly (P=0.03) with age in C57 mice fed a control diet (Figure 4B) and was increased further (P<0.001) when they consumed the high fat diet (Figure 4B). The high fat diet also significantly increased adipocyte content between 24 and 40-week-old mdx mice (Figure 4B). 

Blood serum CK levels are always lower (approximately 10-fold) in C57 mice compared to mdx mice [18] [54] [55] and were not measured for C57 mice in this study. For the mdx mice there was no change in serum CK level between 24 and 40 weeks of age (24 week 3171 ^+^/_- _460 U/L vs. 40 week 2791 ^+^/_- _422 U/L) and no change in CK levels were observed after consuming either a high fat or high protein diet for both 24 and 40-week-old mdx mice (data not shown).  

### Gene expression 

**Group 1) 8 – 24-week-old sedentary mice.**  The mRNA levels of IL1β, but not TNF or IL-6,  was significantly (P=0.05) elevated in the gastrocnemius muscle from 24-week-old mdx compared to C57 mice (Figure 5B). Consumption of the high fat diet significantly increased the mRNA levels of IL1β and IL-6 in C57 mice (Figure 5A, B), but did not alter the levels present in 24-week-old mdx mice. Levels of mRNA for PPAR alpha (P<0.01), PPAR delta/beta (P<0.01) and PGC-1α alpha (P=0.02) (but not PPAR gamma) were significantly decreased (approximately 2 fold) in the gastrocnemius muscle from 24-week-old mdx compared to C57 mice (Figure 6A, C, D). Expression of the three PPARs was unchanged in either strain by consumption of the high fat diet. However, PGC-1α was reduced significantly (P=0.02) in 24-week-old C57 mice fed the high fat compared to the control diet.  

**Figure:**
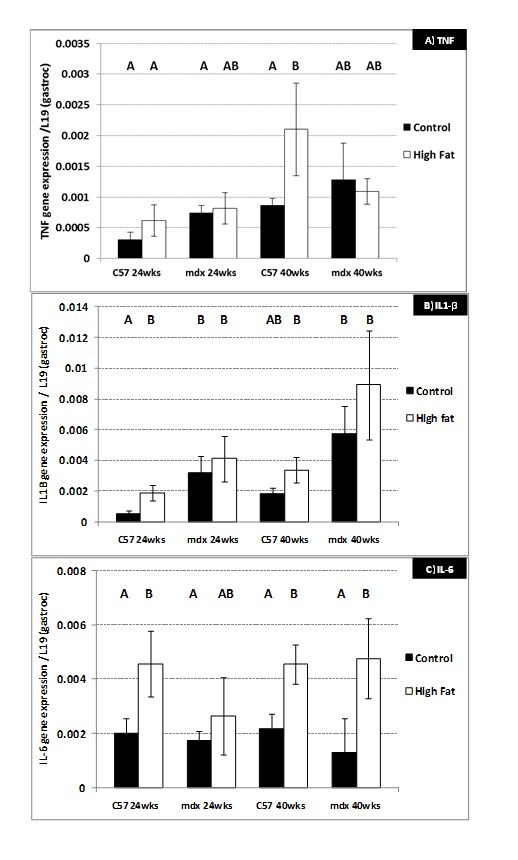
**Figure 5. Gene expression (mRNA) changes in the gastrocnemius muscle of 24 and 40-week-old C57 and mdx mice;** a comparison of sedentary mice on a control diet with mice on a high fat diet. (A) Tumour Necrosis Factor (TNF). (B) Interleukin 1β (IL-1β). (C) Interleukin 6 (IL-6). N= 8 mice per group. A,B,C denotes significant differences, groups with different letters are significantly different from each other (P<0.05).

**Figure:**
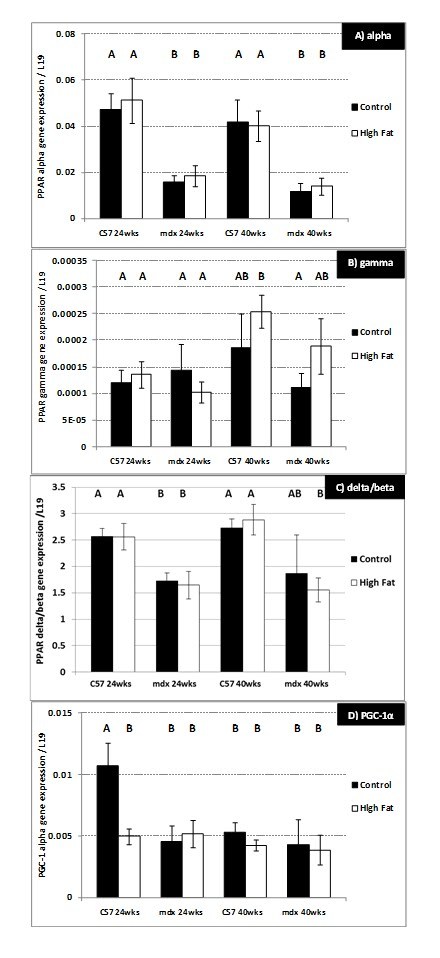
**Figure 6.** **Gene expression (mRNA) changes in the gastrocnemius muscle of 24 and 40-week-old C57 and mdx mice;** a comparison of sedentary mice on a control diet with mice on a high fat diet. (A) Peroxisome proliferator-activated receptor (PPAR) alpha. (B) PPAR gamma. (C) PPAR delta/beta. (D) Peroxisome proliferator-activated receptor gamma coactivator 1 alpha (PGC1-α). N= 8 mice per group. A,B denotes significant differences, groups with different letters are significantly different from each other (P<0.05).

**Group 2) 24 – 40-week-old sedentary mice. **There were no significant changes in inflammatory cytokine mRNA levels with age in either strain of mice fed the control diet. The high fat diet significantly increased TNF (P=0.05) and IL-6 (P=0.05) mRNA in 40-week-old C57 mice and IL-6 mRNA (P=0.04) in mdx mice (Figure 5A, C). mRNA levels of PGC-1α decreased significantly (P=0.05) between 24 and 40-week-old C57 mice (Figure 5D). PPAR alpha mRNA was approximately twofold lower (P=0.02) in the gastrocnemius muscle of 40-week-old mdx mice compared to C57 mice (Figure 5A). The high fat diet caused no change in the mRNA levels of the 3 PPARs or PGC-1α in either strain of 40-week-old mice.  

 **Group 3) 8 – 12-week-old voluntarily exercised mice.**Mdx mice were voluntarily exercised for 4 weeks to increase the low level of dystropathology in skeletal muscles ^[^ ^1^ ^,^ ^50^ ^,^ ^54^ ^]^. Increasing the extent of dystropathology allowed for further evaluation of the potential beneficial effects of a high protein or high fat diet in mdx mice. 

### Food consumption 

There was no significant difference in food intake (g/ d) across the 3 custom diets in both unexercised and exercised mdx mice; however, voluntary wheel exercise significantly increased (P>0.02) food intake (g/ d) for all 3 custom diets by approximately 30% (data not shown). Again, while no significant differences in bodyweight-adjusted average daily food intakes [g/ (g bw .d)] were seen across the 3 custom diets, consumption of all 3 diets was significantly increased (P<0.04) by voluntary exercise (Figure 7A). Weight-adjusted energy intake [kJ /(g bw .d)] was unchanged in sedentary 8 – 12-week-old mdx mice across the 3 diets (Figure 7B) but was increased significantly in exercised mdx mice. Voluntarily exercised mdx mice on the high fat diet had a higher weight-adjusted energy intake [kJ /(g bw .d)] than exercised mdx mice on either the control or the high protein diet (Control Ex, 1.91 **^+^**/**_- _**0.08 [kJ/(g bw .d)]vs. HF Ex 2.28 **^+^**/**_- _**0.08 [kJ/(g bw. d)] ) (Figure 7B).  Absolute energy intake (kJ /day) was also significantly greater in exercised mice on the high fat diet (Control/ Ex, 50.4 **^+^**/**_- _**4.2kJ vs. HF Ex 63.7 **^+^**/**_- _**7.6 kJ). 

**Figure:**
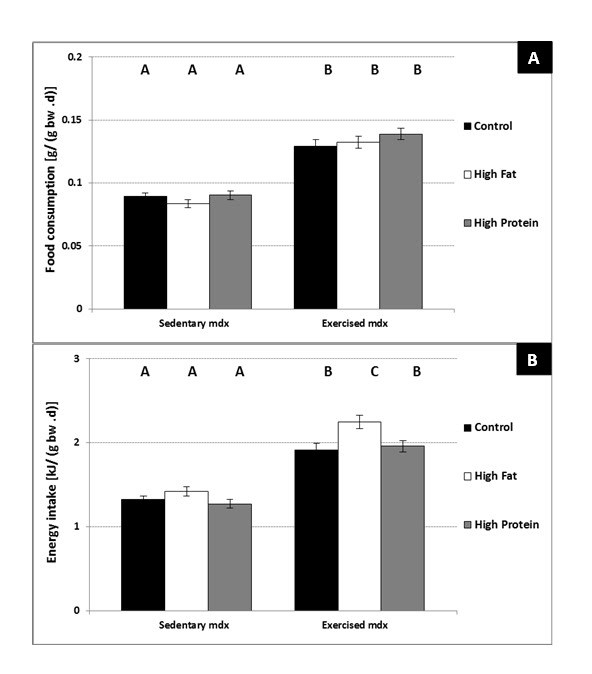
**Figure 7. Average daily food consumption [g/ (g bw .d)] (A) and energy intake [kJ/ (g bw .d)] (B) for mdx mice. **Data for dietary consumption between 8-12 weeks of age is shown for both sedentary and voluntarily exercise mdx mice on either a control diet, high fat diet or a high protein diet. Bars represent standard error. N= 8 for all groups. A,B,C denotes significant differences, groups with different letters are significantly different from each other (P<0.05).

## Body composition.

Voluntary wheel exercise for 4 weeks caused no change in the body weight of 12-week-old mdx mice, and neither did consumption of a high protein or high fat diet (Figure 8A). Exercising the 8 – 12-week-old mdx mice significantly (P=0.03) reduced the epididymal fat pad weight (g fat/ g bw), compared to sedentary mdx mice on a control diet (Figure 8B).  Neither the high protein nor the high fat diet had any significant effect on the epididymal fat pad weight in sedentary 12-week-old mdx mice; however the epidiymal fat pad weight in exercised mdx mice on the high fat diet was increased (P=0.045) compared to those fed the control diet (Figure 7B). No significant change in standardised or absolute gastrocnemius muscle weight was seen after voluntary exercise or diet change (Figure 8C).

**Figure:**
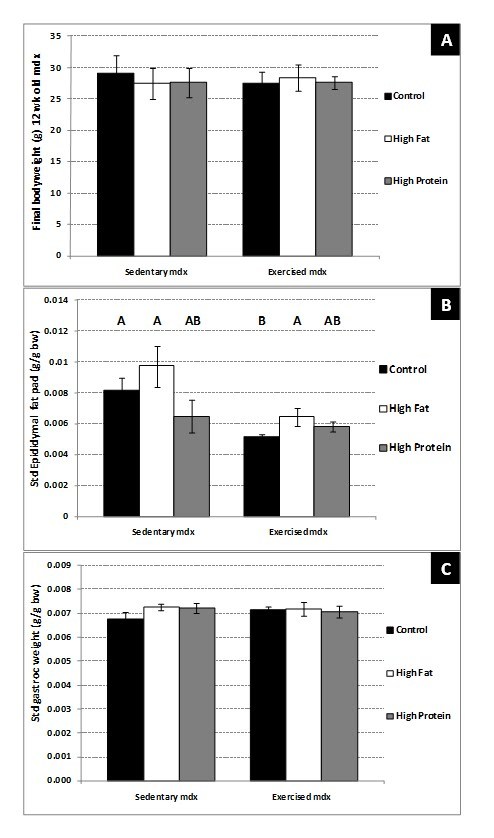
**Figure 8. Body composition of 12-week-old sedentary and voluntarily exercised mdx mice; **a comparison of mice on a control diet with a high fat diet or high protein diet. (A) Total body weight. (B) Standardised epidiymal fat pad weight (g fat /g bw). (C) Standardised gastrocnemius muscle weight (g muscle /g bw). Bars represent standard error. N= 8 for all groups. A,B denotes significant differences, groups with different letters are significantly different from each other (P<0.05).

### Dystropathology

Exercise was completely voluntary and distance run is an indirect indicator of muscle function and exercise capacity. Mdx mice fed the high fat diet ran approximately 50% further (P=0.003) during 4 weeks of exercise (compared to mdx mice on the control diet (Figure 9A). There was no difference in distance run by mdx mice fed the high protein diet compared to the control diet.  

**Figure:**
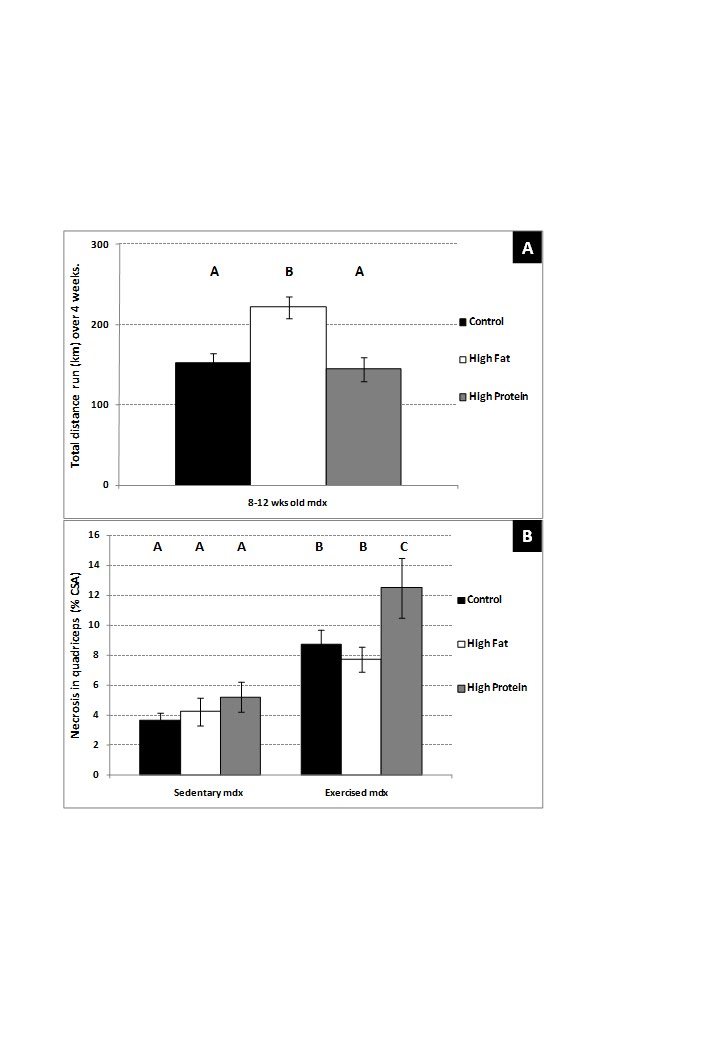
**Figure 9. Total distance run (km) over 4 weeks of voluntary exercise and myofibre necrosis in sedentary and exercised mdx 12-week-old mice; **a comparison of mice on a control diet, high fat diet or high protein diet. (A) Total distance run (km) over 4 weeks of voluntary exercise (mdx only). (B) Myofibre necrosis in the quadriceps muscle (mdx only). Bars represent standard error. N= 8 for all groups. A,B,C denotes significant differences, groups with different letters are significantly different from each other (P<0.05).

Voluntary exercise increased (P=0.02) myofibre necrosis (% CSA) in the quadriceps muscle from 12-week-old mdx mice approximately twofold, relative to the amount present in sedentary mice. The high fat diet for 4 weeks had no effect on myofibre necrosis in either voluntarily exercised or sedentary mdx mice. The high protein diet had no effect on sedentary mice, but significantly (P=0.04) increased myofibre necrosis in exercised mdx mice) (Figure 9B). 

Blood serum CK level was about threefold higher (P=0.04) in voluntarily exercised compared to sedentary 12-week-old mdx mice (sed 4697 ^+^/_- _1898 U/L vs. ex 12913 ^+^/_- _4537 U/L). The CK levels in mdx mice were not affected by a high protein or high fat diet (data not shown).  

Exercise did not change the adipocyte content of the quadriceps muscle from 12-week-old mdx mice (sed mdx 0.54% ^+^/_- _0.07 vs. ex mdx 0.43% ^+^/_- _0.07), nor was there any change in adipocyte content after consuming a high protein or high fat diet in sedentary or exercised mice (data not shown).   

### Gene expression 

Voluntary exercise (for 4 weeks) and/or a high fat diet caused no significant change in the mRNA levels of TNF, IL1β or IL-6 in 12-week-old mdx mice (data not shown). Consumption of the high fat diet had no significant effect on mRNA levels of the 3 PPARs or PGC-1α in sedentary 12-week-old mdx mice; however the combination of voluntary exercise and a high fat diet increased approximately three fold the mRNA levels of PPAR alpha (P<0.01), PPAR gamma (P=0.04), PPAR delta/beta (P<0.01), and PGC-1α (P<0.01) (Figure 10A-D).  

**Figure:**
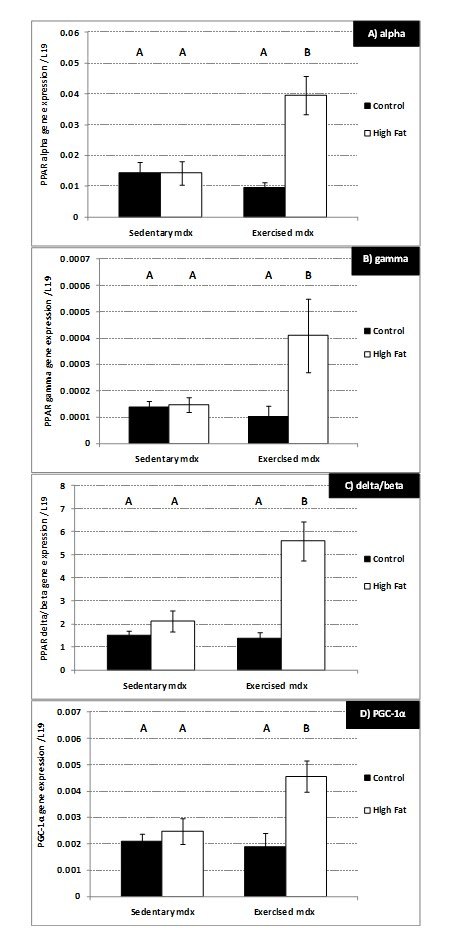
**Figure 10.** **Gene expression (mRNA) changes in the gastrocnemius muscle of 12-week-old sedentary and voluntarily exercised mdx mice;** a comparison of sedentary mice on a control diet with mice on a high fat diet. (A) PPAR alpha. (B) PPAR gamma. (C) PPAR delta/beta. (D) PGC1-α. N= 8 mice per group. A,B denotes significant differences, groups with different letters are significantly different from each other (P<0.05).

## Discussion

**Sedentary mdx and C57 mice.**

To our knowledge, the response of adult mdx mice to a high fat diet has not been described previously. Furthermore, while it is widely documented that C57Bl/6J mice are highly susceptible to diet induced obesity (obesity-prone) [56], it appears that the effects of a high fat diet on C57Bl/10 mice have not been reported.  It is commonly accepted that obesity occurs when there is a chronic positive imbalance between energy intake and energy expenditure, and that long-term consumption of a high fat diet increases deposition of adipose tissue and eventually leads to obesity in both sedentary laboratory rodents and humans [Reviewed in [46]]. Obesity is associated with numerous changes in cell signalling that are believed to underlie much of the morbidity associated with the condition [Reviewed in [46] [57] [58]].

Dystrophic mdx mice were significantly heavier than control C57 mice at both 8 and 24 weeks of age (Figure 2A), primarily due to a well described increase in muscle mass [52] [55] [59] [60] [61] [62] [63]. In contrast, 40-week-old mdx mice were the same weight as C57 mice; this equalising in bodyweight occurred at approximately 32 weeks of age (data not shown) and was most likely due to an increase in body fat in C57 mice (Figure 2B). The body weight of mdx mice did not increase between 24 and 40 weeks of age, nor was there a change in relative epididymal fat pad and gastrocnemius muscle weights (Figure 2A), supporting reports that there is a  stabilisation of the dystrophic phenotype in adult mdx mice [18] [52]. Both 24 and 40-week-old mdx were very lean, with significantly smaller epididymal fat pads, larger gastrocnemius muscles and larger myofibres CSA compared to C57 mice (Figures 2B, 2C, 3). 

The muscular body composition of mdx mice (<40 weeks) is in stark contrast to DMD patients who can move between the spectra of over-nutrition (and obesity) to under-nutrition within their shortened lifespan, and who lose muscle mass at a rate of 4% per year as adults [7] [34] [64]. It is important to consider that the striking loss of muscle mass in DMD patients occurs during the growth phase of the boys (approximately 20 years). In mice however, the damaging main growth phase is exceedingly short by comparison (approximately 6 weeks) [18] [20] [65] with a reduction in myofibre necrosis occurring after growth has ceased in mdx mice. 

We observed significant myofibre hypertrophy in 24-week-old mdx mice (compared to all C57 and 12-week-old mdx mice); however, dystrophic myofibres do not continue to hypertrophy indefinitely, and by 40 weeks of age myofibre CSA was similar to that of 12-week-old mdx mice (Figure 3). We propose that the reduced myofibre size in 40-week-old mdx mice is probably due to myofibre splitting or branching. Shavlakadze et al (2010) reported no difference in myofibre CSA in the quadriceps muscle between 12 and 52 weeks due to significant myofibre splitting in 52-week-old mdx mice [52].  Since our study examined myofibre CSA at 24 weeks and identified significant myofibre hypertrophy in mdx mice at this age, it seems that myofibre splitting occurs sometime beyond 24 weeks, but before 40 weeks of age. The body composition of mdx mice changed between 12 and 24 weeks of age, with significant increases in body weight, absolute and standardised gastrocnemius muscle weight and individual myofibre CSA (Figures 2A, 3, 8A). 

The 8 – 24-week-old mdx mice consumed similar amounts of food and energy compared to age-matched C57 mice across the 3 diets (Figure 1A-C). Yet, there was a large difference in the way the two strains of mice responded to the high fat diet. In contrast to the C57 mice, the mdx mice <24 weeks of age did not deposit significant body fat. This difference between controls and mdx mice in dietary energy utilization may reflect a difference in maintenance energy requirements, with the mdx mice expending more energy for cellular metabolism, including for myofibre hypertrophy and ongoing myogenesis/regeneration, than controls in which dietary fat appears to have been channelled preferentially to fat deposition. 

Between 24 and 40 weeks of age, there was no change in bodyweight (Figure 2A) or gastrocnemius muscle weight (absolute or standardised) and the average myofibre CSA was significantly reduced (Figure 3) for mdx mice on the control diet. The older mdx mice (24-40 weeks) were susceptible to the negative effects of a high fat diet, showing significant increases in total body weight and standardised fat pad weight compared to those on the control diet (Figures 2A, B). This altered response presumably is due to the increase in energy intake (Figure 1B, C), without the same level of muscle damage/regeneration an high energy demanding process, seen in the young mdx mice; as suggested by the progressive reduction of myofibre necrosis in old mdx mice (6% myofibre necrosis in 24 day old mdx mice [1] reducing to approximately 2% by 40 weeks of age) and an absence of continued muscle weight gain.  

In contrast to mdx mice, after consumption of a high fat diet (for 16 weeks) C57 male mice at both 24 and 40 weeks had significantly greater body weights most likely due to a gain in epididymal fat pad weight (and presumably other body fat) (Figure 2A, B) with significant accumulation of adipocytes in the quadriceps muscle of 40-week-old mice (Figure 4B). The increase in energy intake (kJ/day) in 8 - 24-week-old C57 mice on the high fat diet was only minor compared to mice on the control diet (Figure 1B), but when accumulated over 16 weeks could have been enough to produce the observed changes in body composition. However, the increase in energy intake (kJ/day) in the older 24 – 40-week-old C57 mice on the high fat diet was significantly more compared to mice on the control diet (Figure 1B). 

In contrast to the high fat diet (16% fat, 19.4% protein), the high protein diet (50% protein, 7% fat) had little impact on the body composition of 8 – 40-week-old C57 and mdx mice. Initially these two diets were designed to be isocaloric based on digestible energy content (HP, 18.2 MJ/kg vs. HF, 18.1 MJ/Kg) (Table 1). However, there were large differences in the metabolisable energy density of the high fat and high protein diets (HP – 14.09 MJ/kg vs. HF – 16.96 MJ/kg) and this difference produced significant differences in metabolisable energy intake which, in turn, contributed to the different body growth and composition responses to the two diets. A high protein diet or supplementation with certain amino acids (such as leucine) can stimulate skeletal muscle protein synthesis in both young and adult animals, but there is a threshold of protein requirement for maximum growth and once reached, additional protein intake does not further stimulate protein synthesis in skeletal muscle [Reviewed in [66]. Therefore, it is possible that in well-nourished sedentary mice (with adequate protein intake), the high protein diet had no effect on muscle mass. Additionally, the utilization of dietary protein for anabolic processes is dependent on the adequacy of energy intake. If the energy requirements of the mdx mice were increased, and not met by a corresponding increase in intake, dietary amino acids would be preferentially oxidized for energy rather than used for protein synthesis. While it is possible that a beneficial response to high protein consumption might be seen in combination with resistance exercise, such demanding exercise is usually not recommended for dystrophic boys or animal models of DMD due to additional damage to dystrophic muscles. 

Our initial hypothesis was that a high protein and/or high fat diet would help to maintain myofibre integrity and thus reduce dystropathology in mdx mice. Consumption of either diet for 16 weeks did not reduce levels of serum CK (an indicator of myofibre leakiness), even though the high fat diet reduced by approximately 2.5-fold myofibre necrosis in the quadriceps muscle of 24-week-old mdx mice. It is possible that a high fat diet (and increased energy intake) assists mdx mice metabolically and thus helps them to maintain skeletal muscle structure and mass, or that increased lipid coming from the high fat diet stabilises fragile membrane lipids and prevents myofibre necrosis. 

The lack of any benefits with the high protein diet contrasts with studies in mdx mice that show mildly reduced dystropathology after dietary supplementation of creatine, taurine and glutamine (both alone and in combination with prednisolone) [8] [25] [37] [38] [40]. Taurine is not a substrate for protein synthesis, but may improve dystropathology via regulation of calcium homeostasis [67]. Glutamine also has many functions in addition to being a substrate for protein synthesis; for example, it is a precursor for nucleotide synthesis; it is the major energy substrate for rapidly dividing cells such as those of the immune system; and it increases expression of genes that benefit nutrient metabolism and cell survival, including ornithine decarboxylase, heat-shock proteins, and NO synthase [68]. Amino acid supplementation is already widely used amongst DMD patients despite there being no conclusive supporting evidence in the literature [7] [12]. It is important to consider possible counteracting effects when combined interventions are administered. For example, our data from mdx mice on the high protein diet (which contains increased concentrations of the essential amino acids such as leucine and phenylalanine used in protein synthesis, but without an increase in metabolisable energy intake - Table 1) do not support such an intervention since elevated myofibre necrosis was observed in response to voluntarily wheel exercise.

A prolonged positive imbalance between energy intake and expenditure resulting in obesity, is associated with chronic inflammation and may be a potential mechanism by which obesity leads to insulin resistance (in both humans and mice) [Reviewed in [44] [46] [69].  Expression (level of mRNA) of 3 major pro-inflammatory cytokines, TNF, IL1β and I-L6 was up-regulated in the gastrocnemius muscle of both 24 and 40-week-old C57 mice after consumption of the high fat diet (Figure 5A-C).  These increases in gene expression were not seen in mdx mice (Figure 5A-C) which demonstrates, in addition to the lack of changes in body composition, the resistance of mdx mice (particularly <24 weeks) to a high fat diet. Inflammation plays a major role in myofibre necrosis and regeneration in skeletal muscle, thus the inflammatory state can be an indirect indicator of the extent of dystropathology [1] [42]. 

The high fat diet significantly reduced PGC-1α gene expression in 24-week-old C57 mice but, apart from this, there were no other significant changes in PPAR expression caused by the high fat diet in either strain of mice. Compared with C57 mice, the levels of PGC-1α, PPAR alpha and PPAR delta/beta were all much lower (approximately half) in mdx mice on a control diet; again this endorses that metabolic processes are altered in dystrophic muscles of mdx mice.  

Due to the multiple systemic effects that the 3 PPARs and PGC-1α have on whole body metabolism (e.g. lipid metabolism) [46] [48] [70] we assume that decreased gene expression of the 3 PPARs and PGC-1α in dystrophic skeletal muscle would have a wide range of effects. However, the precise consequence(s) in dystrophic skeletal muscle, to our knowledge, are not documented. Decreased PGC-1α expression in skeletal muscles of C57Bl/6J mice decreases exercise capacity and fatigue resistance [Reviewed in [48]] and low levels of PGC-1α mRNA in mdx mice on a control diet, correspond with decreased exercise capacity and increased fatigue that are common features of dystrophic muscle [18] [51] [71]. It has also been shown that several gene programmes linked to PGC-1α are dysregulated in dystrophic muscle (e.g. mitochondrial function, calcium handling and ROS production) and introduction of a muscle specific PGC-1α transgene into mdx mice improved muscle function and structure, possibly via up-regulation of utrophin [72] [73]. Overall there seems to be an association with decreased PGC-1α expression having detrimental effects in both dystrophic and normal muscle. Although the combined consequences of PGC-1α, PPAR alpha and PPAR delta/beta in dystrophic (compared with normal) muscle are not clear, they all increased in mdx muscles in response to voluntary exercise combined with a high fat diet (see below) with beneficial effects.

**Voluntarily exercised mice.**

In order to further examine the potential benefits of a high fat or high protein diet on dystrophic muscle, 8 – 12-week-old mdx mice were voluntarily exercised to exacerbate the dystropathology [1] [49] [50] [54]. Although the 12-week-old mdx mice were very lean, there was a further reduction in epididymal fat pad weight after 4 weeks of voluntary exercise (Figure 8B). It appears that mdx mice were able to maintain body weight and muscle mass (Figure 8A, C) over 4 weeks of voluntary exercise by increasing food consumption and energy intake (Figure 7A, B). 

Despite a high fat diet reducing myofibre necrosis in sedentary 24-week-old mdx mice (Figure 4), this diet had no significant benefit on myofibre necrosis or serum CK levels in voluntarily exercised mdx mice. Energy intake was significantly increased in voluntarily exercised mdx on the high fat diet compared to mdx mice on the control diet (Figure 7B). This may have enabled the mdx mice to run further (approximately 50%) without producing further muscle damage (e.g. myofibre necrosis or serum CK level). A possible interpretation is that the high fat diet was able to better meet the increased energy needs of the mdx mice thereby enabling them to run further while also maintaining myofibre integrity.  

A striking increase in the mRNA levels of PPAR alpha, PPAR gamma, PPAR delta/beta and PGC-1α was seen in the gastrocnemius muscle of exercised mdx mice on the high fat diet.  Exercise is known to increase PGC-1α expression due to increased neuromuscular input and elevated levels of MEF2 and CREB expression with exercise [48] , and increases in PGC-1α and PPAR delta/beta are both beneficial to mdx dystropatholgy *in vivo* [73] [74]. Therefore, it is proposed that the increased capacity for exercise in dystrophic mdx mice fed a high fat diet may be modulated, in part, via up-regulation of the 3 PPARs and PGC-1α. 

## **Conclusion**

This research clearly identifies an ‘altered’ response to a high fat diet in dystrophic muscles of mdx mice compared to C57 controls. This response was pronounced in younger mdx mice <24 weeks old, but diminished with age (by 40 weeks). The high fat diet appears to have enabled the mdx mice to better meet their energy needs, and reduced the severity of their dystropathology and increased their voluntary exercise ability. The high protein diet had no significant effects on the body composition of either strain of mice and no benefit on mdx dystropathology; this may be due, in part, to its lower metabolisable energy density compared with the high fat diet, resulting in an energy intake that was not sufficient to promote maximal utilization of the increased protein availability. 

Our new data highlight the potential benefits of a high fat diet on dystrophic skeletal muscle. This study in mdx mice raises many interesting questions about possible differences in metabolism and energy demands between dystrophic and normal muscle. The extent to which this benefit of a high fat diet is due to an increased energy intake *per se, *or to specific benefits of increased dietary fat directly on dystrophic muscles is not yet clear: determining the precise mechanism responsible for these demonstrated benefits is an important future goal. In addition, the effects of a high fat diet on dystrophic heart function and pathology needs to be addressed in mdx mice, before considering translation to the clinical situation. It is appreciated that in corticosteroid-treated inactive DMD patients, a high fat diet may contribute to obesity and thus impact on disease severity (especially since corticosteroid usage is known to contribute to obesity and Cushingoid symptoms). In addition, the impact of a high fat diet might be different in steroid-naïve DMD boys and also influenced by growth and disease severity. Clearly, very careful management is required to achieve a fine balance between the increased metabolic demands of dystrophic muscle at different stages of the disease, possible interactions with other drugs, and the potential negative consequences of chronically consuming a high fat diet

## Acknowledgements

The authors thank Mr Greg Cozens (School of Anatomy & Human Biology, UWA) for excellent technical assistance, Dr Andrew Hoey (University of Southern Queensland) for consultation regarding the preliminary studies and Mr Warren Potts (Specialty Feeds, WA) for design and manufacture of the three semi-purified diets. Research funding from the Australian National Health and Medical Research Council (MG) and Australian Postgraduate Award Scholarships (HR-C) are gratefully acknowledged.

## Funding information

This research is funded by the Australian National Health and Medical Research Council (MG) and Australian Postgraduate Award Scholarships (HR-C).

## Competing interests

The authors have declared that no competing interests exist. 

## References

[ref-961947258] Radley HG, Davies MJ, and Grounds MD. Reduced muscle necrosis and long-term benefits in dystrophic mdx mice after cV1q (blockade of TNF) treatment. Neuromuscul Disord. 2008; 18: 227-38. 10.1016/j.nmd.2007.11.002 18207402

[ref-1924831528] Tidball JG and Wehling-Henricks M. The role of free radicals in the pathophysiology of muscular dystrophy. J Appl Physiol. 2007; 102: 1677-86. 10.1152/japplphysiol.01145.2006 17095633

[ref-1159087936] Whitehead NP, Pham C, Gervasio OL, and Allen DG. N-Acetylcysteine ameliorates skeletal muscle pathophysiology in mdx mice. J Physiol. 2008; 586: 2003-14. 10.1113/jphysiol.2007.148338 PMC2375717 18258657

[ref-106965215] Kuznetsov AV, Winkler K, Wiedemann FR, von Bossanyi P, Dietzmann K, and Kunz WS. Impaired mitochondrial oxidative phosphorylation in skeletal muscle of the dystrophin-deficient mdx mouse. Mol Cell Biochem. 1998; 183: 87-96. 10.1023/a:1006868130002 9655182

[ref-2876130824] Even PC, Decrouy A, and Chinet A. Defective regulation of energy metabolism in mdx-mouse skeletal muscles. Biochem J. 1994; 304 ( Pt 2): 649-54. 10.1042/bj3040649 PMC1137540 7999003

[ref-3175993803] Evans NP, Misyak SA, Robertson JL, Bassaganya-Riera J, and Grange RW. Dysregulated intracellular signaling and inflammatory gene expression during initial disease onset in Duchenne muscular dystrophy. Am J Phys Med Rehabil. 2009; 88: 502-22. 10.1097/PHM.0b013e3181a5a24f 19454857

[ref-2846275368] Davidson ZE and Truby H. A review of nutrition in Duchenne muscular dystrophy. J Hum Nutr Diet. 2009; 22: 383-93. 10.1111/j.1365-277X.2009.00979.x 19743977

[ref-3608082636] Radley HG, De Luca A, Lynch GS, and Grounds MD. Duchenne muscular dystrophy: Focus on pharmaceutical and nutritional interventions. Int J Biochem Cell Biol. 2007; 39: 469-77. 10.1016/j.biocel.2006.09.009 17137828

[ref-2986508416] Manzur AY, Kuntzer T, Pike M, and Swan A. Glucocorticoid corticosteroids for Duchenne muscular dystrophy. Cochrane database of systematic reviews (Online). 2008; CD003725. 10.1002/14651858.CD003725.pub3 18254031

[ref-3645251579] Angelini C. The role of corticosteroids in muscular dystrophy: a critical appraisal. Muscle Nerve. 2007; 36: 424-35. 10.1002/mus.20812 17541998

[ref-2287746707] Biggar WD, Harris VA, Eliasoph L, and Alman B. Long-term benefits of deflazacort treatment for boys with Duchenne muscular dystrophy in their second decade. Neuromuscul Disord. 2006; 16: 249-255. 10.1016/j.nmd.2006.01.010 16545568

[ref-2107796700] Bushby K, Finkel R, Birnkrant DJ, Case LE, Clemens PR, Cripe L, Kaul A, Kinnett K, McDonald C, Pandya S, Poysky J, Shapiro F, Tomezsko J, and Constantin C. Diagnosis and management of Duchenne muscular dystrophy, part 1: diagnosis, and pharmacological and psychosocial management. Lancet Neurol. 2010; 9: 77-93. 10.1016/S1474-4422(09)70271-6 19945913

[ref-715223638] Pichavant C, Aartsma-Rus A, Clemens PR, Davies KE, Dickson G, Takeda S, Wilton SD, Wolff JA, Wooddell CI, Xiao X, and Tremblay JP. Current status of pharmaceutical and genetic therapeutic approaches to treat DMD. Molecular therapy : the journal of the American Society of Gene Therapy. 2011; 19: 830-40. 10.1038/mt.2011.59 PMC3098643 21468001

[ref-532549463] Wells DJ. Treatments for muscular dystrophy: increased treatment options for Duchenne and related muscular dystropies. Gene Ther. 2008; 15: 1077-8. 10.1038/gt.2008.97 18528429

[ref-1081405737] Guglieri M and Bushby K. Molecular treatments in Duchenne muscular dystrophy. Curr Opin Pharmacol. 2010. 10.1016/j.coph.2010.03.005 20434401

[ref-3666744332] Manzur AY and Muntoni F. Diagnosis and new treatments in muscular dystrophies. J Neurol Neurosurg Psychiatry. 2009; 80: 706-14. 10.1136/jnnp.2008.158329 19531685

[ref-3018146969] Nagaraju K and Willmann R. Developing standard procedures for murine and canine efficacy studies of DMD therapeutics: report of two expert workshops on "Pre-clinical testing for Duchenne dystrophy": Washington DC, October 27th-28th 2007 and Zurich, June 30th-July 1st 2008. Neuromuscul Disord. 2009; 19: 502-6. 10.1016/j.nmd.2009.05.003 PMC2766092 19560356

[ref-2122416613] Grounds MD, Radley HG, Lynch GS, Nagaraju K, and De Luca A. Towards developing standard operating procedures for pre-clinical testing in the mdx mouse model of Duchenne muscular dystrophy. Neurobiol Dis. 2008; 31: 1-19. 10.1016/j.nbd.2008.03.008 PMC2518169 18499465

[ref-4206556478] Vainzof M, Ayub-Guerrieri D, Onofre PC, Martins PC, Lopes VF, Zilberztajn D, Maia LS, Sell K, and Yamamoto LU. Animal models for genetic neuromuscular diseases. J Mol Neurosci. 2008; 34: 241-8. 10.1007/s12031-007-9023-9 18202836

[ref-2633419718] Grounds MD. Two-tiered hypotheses for Duchenne muscular dystrophy. Cell Mol Life Sci. 2008; 65: 1621-5. 10.1007/s00018-008-7574-8 PMC11131677 18327663

[ref-4155370825] Willmann R, Possekel S, Dubach-Powell J, Meier T, and Ruegg MA. Mammalian animal models for Duchenne muscular dystrophy. Neuromuscul Disord. 2009; 19: 241-9. 10.1016/j.nmd.2008.11.015 19217290

[ref-546042920] El-Shafey AF, Armstrong AE, Terrill JR, Grounds MD, and Arthur PG. Screening for increased protein thiol oxidation in oxidatively stressed muscle tissue. Free radical research. 2011; 45: 991-9. 10.3109/10715762.2011.590136 21696323

[ref-605056544] Dupont-Versteegden EE, Baldwin RA, McCarter RJ, and Vonlanthen MG. Does muscular dystrophy affect metabolic rate? Journal of Neurological Sciences. 1994; 121: 203-207. 10.1016/0022-510x(94)90353-0 8158216

[ref-3980324342] Landisch RM, Kosir AM, Nelson SA, Baltgalvis KA, and Lowe DA. Adaptive and nonadaptive responses to voluntary wheel running by mdx mice. Muscle Nerve. 2008; 38: 1290-303. 10.1002/mus.21141 PMC3392332 18816601

[ref-2289124511] Passaquin AC, Renard M, Kay L, Challet C, Mokhtarian A, Wallimann T, and Ruegg UT. Creatine supplementation reduces skeletal muscle degeneration and enhances mitochondrial function in mdx mice. Neuromuscul Disord. 2002; 12: 174-182. 10.1016/s0960-8966(01)00273-5 11738360

[ref-1150883093] Han R, Grounds MD, and Bakker AJ. Measurement of sub-membrane [Ca(2+)] in adult myofibers and cytosolic [Ca(2+)] in myotubes from normal and mdx mice using the Ca(2+) indicator FFP-18. Cell Calcium. 2006; 40: 299-307. 10.1016/j.ceca.2006.04.016 16765438

[ref-2224961592] Whitehead NP, Yeung EW, and Allen DG. Muscle damage in mdx (dystrophic) mice: role of calcium and reactive oxygen species. Clin Exp Pharmacol Physiol. 2006; 33: 657-662. 10.1111/j.1440-1681.2006.04394.x 16789936

[ref-2994327432] MacLennan PA and Edwards RH. Protein turnover is elevated in muscle of mdx mice in vivo. Biochem J. 1990; 268: 795-7. 10.1042/bj2680795 PMC1131511 2194450

[ref-1351279693] Griggs RC and Rennie MJ. Muscle wasting in muscular dystrophy: decreased protein synthesis or increased degradation? Ann Neurol. 1983; 13: 125-32. 10.1002/ana.410130204 6338807

[ref-4089555816] Zanardi MC, Tagliabue A, Orcesi S, Berardinelli A, Uggetti C, and Pichiecchio A. Body composition and energy expenditure in Duchenne muscular dystrophy. Eur J Clin Nutr. 2003; 57: 273-8. 10.1038/sj.ejcn.1601524 12571659

[ref-3073339658] Helliwell TR, MacLennan PA, McArdle A, Edwards RHT, and Jackson MJ. Fasting increases the extent of muscle necrosis in the mdx mouse. Clinical Science. 1996; 90: 467-472. 10.1042/cs0900467 8697716

[ref-174100161] Bogdanovich Sea. Therapeutics for Duchenne muscular dystrophy: current approaches and future directions. Journal of Molecular Medicine. 2004; 82: 102-115. 10.1007/s00109-003-0484-1 14673527

[ref-3317030322] Pearlman JP and Fielding RA. Creatine monohydrate as a therapeutic aid in muscular dystrophy. Nutrition Review. 2006; 64: 80-8. 10.1301/nr.2006.feb.80-88 16536185

[ref-3479096549] Leighton S. Nutrition for boys with Duchenne muscular dystrophy. Nutrition and Dietetics. 2003; 60: 11 - 15.

[ref-3951285368] Escolar DM, Buyse G, Henricson E, Leshner R, Florence J, Mayhew J, Tesi-Rocha C, Gorni K, Pasquali L, Patel KM, McCarter R, Huang J, Mayhew T, Bertorini T, Carlo J, Connolly AM, Clemens PR, Goemans N, Iannaccone ST, Igarashi M, Nevo Y, Pestronk A, Subramony SH, Vedanarayanan VV, and Wessel H. CINRG randomized controlled trial of creatine and glutamine in Duchenne muscular dystrophy. Ann Neurol. 2005; 58: 151-5. 10.1002/ana.20523 15984021

[ref-614609692] Tarnopolsky MA, Mahoney DJ, Vajsar J, Rodriguez C, Doherty TJ, Roy BD, and Biggar D. Creatine monohydrate enhances strength and body composition in Duchenne muscular dystrophy. Neurology. 2004; 62: 1771-7. 10.1212/01.wnl.0000125178.18862.9d 15159476

[ref-1847212194] De Luca A, Pierno S, Liantonio A, Cetrone M, Camerino C, Fraysse B, Mirabella M, Servidei S, Ruegg UT, and Conte Camerino D. Enhanced dystrophic progression in mdx mice by exercise and beneficial effects of taurine and insulin-like growth factor-1. Journal of Pharmacological and Experimental Therapeutics. 2003; 304: 453-63. 10.1124/jpet.102.041343 12490622

[ref-447571161] Granchelli JA, Pollina C, and Hudecki MS. Pre-clinical screening of drugs using the mdx mouse. Neuromusclular Disorders. 2000; 10: 235-239. 10.1016/s0960-8966(99)00126-1 10838248

[ref-2913133682] Payne ET, Yasuda N, Bourgeois JM, Devries MC, Rodriguez MC, Yousuf J, and Tarnopolsky MA. Nutritional therapy improves function and complements corticosteroid intervention in mdx mice. Muscle Nerve. 2006; 33: 66-77. 10.1002/mus.20436 16149047

[ref-2626541767] Cozzoli A, Rolland JF, Capogrosso RF, Sblendorio VT, Longo V, Simonetti S, Nico B, and De Luca A. Evaluation of Potential Synergistic Action of a Combined Treatment with Alpha-Methyl-Prednisolone and Taurine on the Mdx Mouse Model of Duchenne Muscular Dystrophy. Neuropathol Appl Neurobiol. 2010; 10.1111/j.1365-2990.2010.01106.x 20618838

[ref-272829529] Zdanowicz MM, Slonim AE, Bilaniuk I, O'Connor MM, Moyse J, and Teichberg S. High protein diet has beneficial effects in murine muscular dystrophy. J Nutr. 1995; 125: 1150-8. 10.1093/jn/125.5.1150 7738674

[ref-893390614] Grounds MD, Radley HG, Gebski BL, Bogoyevitch MA, and Shavlakadze T. Implications of cross-talk between tumour necrosis factor and insulin-like growth factor-1 signalling in skeletal muscle. Clin Exp Pharmacol Physiol. 2008; 35: 846-51. 10.1111/j.1440-1681.2007.04868.x 18215180

[ref-757210157] Tidball JG and Wehling-Henricks M. Damage and inflammation in muscular dystrophy: potential implications and relationships with autoimmune myositis. Curr Opin Rheumatol. 2005; 17: 707-713. 10.1097/01.bor.0000179948.65895.1a 16224247

[ref-1855074634] Wisse BE. The inflammatory syndrome: the role of adipose tissue cytokines in metabolic disorders linked to obesity. J Am Soc Nephrol. 2004; 15: 2792-800. 10.1097/01.ASN.0000141966.69934.21 15504932

[ref-439923913] Pedersen BK. IL-6 signalling in exercise and disease. Biochem Soc Trans. 2007; 35: 1295-7. 10.1042/BST0351295 17956334

[ref-1388816574] Stienstra R, Duval C, Muller M, and Kersten S. PPARs, Obesity, and Inflammation. PPAR Res. 2007; 2007: 95974. 10.1155/2007/95974 PMC1783744 17389767

[ref-2964751045] Zierath JR and Hawley JA. Skeletal muscle fiber type: influence on contractile and metabolic properties. PLoS Biol. 2004; 2: e348. 10.1371/journal.pbio.0020348 PMC521732 15486583

[ref-2604456746] Liang H and Ward WF. PGC-1alpha: a key regulator of energy metabolism. Adv Physiol Educ. 2006; 30: 145-51. 10.1152/advan.00052.2006 17108241

[ref-3155620892] Hodgetts S, Radley H, Davies M, and Grounds MD. Reduced necrosis of dystrophic muscle by depletion of host neutrophils, or blocking TNFalpha function with Etanercept in mdx mice. Neuromuscul Disord. 2006; 16: 591-602. 10.1016/j.nmd.2006.06.011 16935507

[ref-2981959707] Radley HG and Grounds MD. Cromolyn administration (to block mast cell degranulation) reduces necrosis of dystrophic muscle in mdx mice. Neurobiol Dis. 2006; 23: 387-97. 10.1016/j.nbd.2006.03.016 16798005

[ref-1811659586] Hara H, Nolan PM, Scott MO, Bucan M, Wakayama Y, and Fischbeck KH. Running endurance abnormality in mdx mice. Muscle Nerve. 2002; 25: 207-11. 10.1002/mus.10023 11870688

[ref-4197118450] Shavlakadze T, Chai J, Maley K, Cozens G, Grounds G, Winn N, Rosenthal N, and Grounds MD. A growth stimulus is needed for IGF-1 to induce skeletal muscle hypertrophy in vivo. J Cell Sci. 2010; 123: 960-71. 10.1242/jcs.061119 20179101

[ref-578956998] Turpin SM, Ryall JG, Southgate R, Darby I, Hevener AL, Febbraio MA, Kemp BE, Lynch GS, and Watt MJ. Examination of 'lipotoxicity' in skeletal muscle of high-fat fed and ob/ob mice. J Physiol. 2009; 587: 1593-605. 10.1113/jphysiol.2008.166033 PMC2678228 19204053

[ref-2524736714] Radley-Crabb H, Terrill J, Shavlakadze T, Tonkin J, Arthur P, and Grounds M. A single 30min treadmill exercise session is suitable for 'proof-of concept studies' in adult mdx mice: A comparison of the early consequences of two different treadmill protocols. Neuromuscular disorders : NMD. 2011. 10.1016/j.nmd.2011.07.008 21835619

[ref-1992041680] Spurney CF, Gordish-Dressman H, Guerron AD, Sali A, Pandey GS, Rawat R, Van Der Meulen JH, Cha HJ, Pistilli EE, Partridge TA, Hoffman EP, and Nagaraju K. Preclinical drug trials in the mdx mouse: Assessment of reliable and sensitive outcome measures. Muscle Nerve. 2009; 39: 591-602. 10.1002/mus.21211 PMC4116326 19260102

[ref-4066761849] Alexander J, Chang GQ, Dourmashkin JT, and Leibowitz SF. Distinct phenotypes of obesity-prone AKR/J, DBA2J and C57BL/6J mice compared to control strains. International journal of obesity (2005). 2006; 30: 50-9. 10.1038/sj.ijo.0803110 16231032

[ref-1053878097] Buettner R, Scholmerich J, and Bollheimer LC. High-fat diets: modeling the metabolic disorders of human obesity in rodents. Obesity (Silver Spring, Md. 2007; 15: 798-808. 10.1038/oby.2007.608 17426312

[ref-516006080] Pedersen BK. Muscle-to-fat interaction: a two-way street? J Physiol. 2010; 588: 21. 10.1113/jphysiol.2009.184747 PMC2821541 20045903

[ref-1865314454] Peter AK and Crosbie RH. Hypertrophic response of Duchenne and limb-girdle muscular dystrophies is associated with activation of Akt pathway. Exp Cell Res. 2006; 312: 2580-91. 10.1016/j.yexcr.2006.04.024 16797529

[ref-69388718] Anderson JE, Ovalle WK, and Bressler BH. Electron microscopic and autoradiographic characterization of hindlimb muscle regeneration in the mdx mouse. Anat Rec. 1987; 219: 243-257. 10.1002/ar.1092190305 3425943

[ref-3227234366] Coulton GR, Curtin NA, Morgan JE, and Partridge TA. The mdx mouse skeletal muscle myopathy: II. Contractile properties. Neuropathol Appl Neurobiol. 1988; 14: 299-314. 10.1111/j.1365-2990.1988.tb00890.x 3221977

[ref-2105583775] Mokhtarian A, Decrouy A, Chinet A, and Even PC. Components of energy expenditure in the mdx mouse model of Duchenne muscular dystrophy. Pflugers Arch. 1996; 431: 527-32. 10.1007/BF02191899 8596695

[ref-3816658693] Connolly AM, Keeling RM, Mehta S, Pestronk A, and Sanes JR. Three mouse models of muscular dystrophy: the natural history of strength and fatigue in dystrophin-, dystrophin/utrophin-, and laminin alpha2-deficient mice. Neuromuscul Disord. 2001; 11: 703-12. 10.1016/s0960-8966(01)00232-2 11595512

[ref-3529495525] Griffiths RD and Edwards RH. A new chart for weight control in Duchenne muscular dystrophy. Arch Dis Child. 1988; 63: 1256-8. 10.1136/adc.63.10.1256 PMC1779007 3196052

[ref-402564773] Grounds MD and Shavlakadze T. Growing muscle has different sarcolemmal properties from adult muscle: a proposal with scientific and clinical implications: reasons to reassess skeletal muscle molecular dynamics, cellular responses and suitability of experimental models of muscle disorders. BioEssays : news and reviews in molecular, cellular and developmental biology. 2011; 33: 458-68. 10.1002/bies.201000136 21500235

[ref-1963278666] Shavlakadze T and Grounds M. Of bears, frogs, meat, mice and men: complexity of factors affecting skeletal muscle mass and fat. BioEssays. 2006; 28: 994-1009. 10.1002/bies.20479 16998828

[ref-1448527324] Conte Camerino D, Tricarico D, Pierno S, Desaphy JF, Liantonio A, Pusch M, Burdi R, Camerino C, Fraysse B, and De Luca A. Taurine and skeletal muscle disorders. Neurochem Res. 2004; 29: 135-42. 10.1023/b:nere.0000010442.89826.9c 14992272

[ref-1778639442] Curi R, Lagranha CJ, Doi SQ, Sellitti DF, Procopio J, Pithon-Curi TC, Corless M, and Newsholme P. Molecular mechanisms of glutamine action. Journal of cellular physiology. 2005; 204: 392-401. 10.1002/jcp.20339 15795900

[ref-4190276528] Arkan MC, Hevener AL, Greten FR, Maeda S, Li ZW, Long JM, Wynshaw-Boris A, Poli G, Olefsky J, and Karin M. IKK-beta links inflammation to obesity-induced insulin resistance. Nat Med. 2005; 11: 191-8. 10.1038/nm1185 15685170

[ref-3562758842] Ehrenborg E and Krook A. Regulation of skeletal muscle physiology and metabolism by peroxisome proliferator-activated receptor delta. Pharmacol Rev. 2009; 61: 373-93. 10.1124/pr.109.001560 19805479

[ref-4086838570] Piers AT, Lavin T, Radley-Crabb HG, Bakker AJ, Grounds MD, and Pinniger GJ. Blockade of TNF in vivo using cV1q antibody reduces contractile dysfunction of skeletal muscle in response to eccentric exercise in dystrophic mdx and normal mice. Neuromuscul Disord. 2011; 21: 132-41. 10.1016/j.nmd.2010.09.013 21055937

[ref-1914335304] Davies KE and Khurana TS. A new way to regulate the NMJ. Nat Med. 2007; 13: 538-9. 10.1038/nm0507-538 17479096

[ref-1261122381] Handschin C, Kobayashi YM, Chin S, Seale P, Campbell KP, and B.M. S. PGC-1alpha regulates the neuromuscular junction program and ameliorates Duchenne muscular dystrophy. Genes Dev. 2007; 21: 770-783. 10.1101/gad.1525107 PMC1838529 17403779

[ref-1733274977] Miura P, Chakkalakal JV, Boudreault L, Belanger G, Hebert RL, Renaud JM, and Jasmin BJ. Pharmacological activation of PPARbeta/delta stimulates utrophin A expression in skeletal muscle fibers and restores sarcolemmal integrity in mature mdx mice. Hum Mol Genet. 2009; 18: 4640-9. 10.1093/hmg/ddp431 19744959

